# RETRACTION: MicroRNA‐425‐3p Inhibits Myocardial Inflammation and Cardiomyocyte Apoptosis in Mice With Viral Myocarditis Through Targeting TGF‐β1

**DOI:** 10.1002/iid3.70190

**Published:** 2025-04-11

## Abstract

**RETRACTION**: J. Li, J. Tu, H. Gao and L. Tang “MicroRNA‐425‐3p Inhibits Myocardial Inflammation and Cardiomyocyte Apoptosis in Mice With Viral Myocarditis Through Targeting TGF‐β1,” *Immunity, Inflammation and Disease* 9, no. 1 (2021): 288–298, https://doi.org/10.1002/iid3.392.

The above article, published online on 17 December 2020 in Wiley Online Library (wileyonlinelibrary.com), has been retracted by agreement between the journal Editor‐in‐Chief, Marc Veldhoen; and John Wiley & Sons Ltd. The retraction has been agreed following an investigation into concerns raised by a third party. The investigation revealed inappropriate duplication of the control and CVB3 images presented in Figure 6A. Additionally, the CVB3 + miR‐425‐3p mimic image shown in Figure 6A was previously published in another article in a different scientific context. Lastly, evidence of undisclosed splicing was observed in the western blots shown in Figures 5F, 6C and 6E. The authors were invited to comment and provide their raw data, but they were unable to supply any data or an explanation. The editors consider the results and conclusion reported in this article unreliable. The authors were informed of the retraction.

**RETRACTION**: J. Li, J. Tu, H. Gao and L. Tang “MicroRNA‐425‐3p Inhibits Myocardial Inflammation and Cardiomyocyte Apoptosis in Mice With Viral Myocarditis Through Targeting TGF‐β1,” *Immunity, Inflammation and Disease* 9, no. 1 (2021): 288–298, https://doi.org/10.1002/iid3.392.

The above article, published online on 17 December 2020 in Wiley Online Library (wileyonlinelibrary.com), has been retracted by agreement between the journal Editor‐in‐Chief, Marc Veldhoen; and John Wiley & Sons Ltd. The retraction has been agreed following an investigation into concerns raised by a third party. The investigation revealed inappropriate duplication of the control and CVB3 images presented in Figure 6A. Additionally, the CVB3 + miR‐425‐3p mimic image shown in Figure 6A was previously published in another article in a different scientific context. Lastly, evidence of undisclosed splicing was observed in the western blots shown in Figures 5F, 6C and 6E. The authors were invited to comment and provide their raw data, but they were unable to supply any data or an explanation. The editors consider the results and conclusion reported in this article unreliable. The authors were informed of the retraction.

